# Narratives or not? Examining the roles of message format and individuals’ stages of change in the context of HPV vaccination promotion

**DOI:** 10.1093/her/cyaf015

**Published:** 2025-05-12

**Authors:** Mengfei Guan, Shawn C Chiang, Regan M Murray, Wen-Juo Lo, Larry T Hill, Ann C Klassen, Jennifer A Manganello, Amy E Leader, Philip M Massey

**Affiliations:** Department of Communication, University of Oklahoma, Burton Hall, 610 Elm Avenue, Norman, OK 73019, USA; Department of Health Behavior, School of Public Health, Texas A&M University, 212 Adriance Lab Rd, College Station, TX 77843, USA; Department of Mental Health, Johns Hopkins Bloomberg School of Public Health, Hampton House, 624 N. Broadway, Baltimore, MD 21205, USA; Department of Counseling, Leadership, and Research Methods, University of Arkansas, 100 Graduate Education Building, Fayetteville, AR 72701, USA; College of Medicine Northwest, University of Arkansas for Medical Sciences, 1125 N College Ave, Fayetteville, AR 72701, USA; Department of Community Health and Prevention, Dornsife School of Public Health, Drexel University, Nesbitt Hall, 3215 Market St, Philadelphia, PA 19104, USA; Department of Health Policy, Management and Behavior, College of Integrated Health Sciences, University at Albany, State University of New York (SUNY), 1 University Place, Rensselaer, NY 12144, USA; Division of Population Science, Department of Medical Oncology, Thomas Jefferson University, 834 Chestnut Street, Philadelphia, PA 19107, USA; Department of Community Health Sciences, UCLA Fielding School of Public Health, 650 Charles E. Young Dr. South, Los Angeles, CA 90095, USA

## Abstract

Narrative persuasion has been widely used in health communication campaigns and persuasive message design. However, several meta-analyses showed that the relative effectiveness of narratives in promoting behavior change was not consistently observed in the existing literature. With the goal of exploring boundary conditions of narrative effects, this study investigates the interaction effects of narrative persuasion and stages of change on promoting behavior change in the context of encouraging parents to vaccinate their children against human papillomavirus (HPV). Findings from an online experiment (*N* = 593) showed that non-narrative messages were more effective in bolstering behavioral intention than narrative messages among people who were not ready to engage in behavior change (i.e. in the precontemplation stage). In addition, among people who were thinking about changing their behavior (i.e. in the contemplation stage) or motivated to take action (i.e. in the preparation stage), both narratives and non-narratives were effective in increasing behavioral intention. This study contributes important theoretical insights to the role of narratives in health communication. Public health professionals may consider tailoring message design strategies to audience characteristics to enhance message effectiveness.

## Introduction

The human papillomavirus (HPV) is the most common sexually transmitted infection in the United States, with around 13 million new HPV infections each year, bringing the total number infected to 42 million [1]. Although most HPV infections clear on their own, some strains of HPV cause cancer. Evidence supporting HPV vaccine efficacy and safety is robust, but coverage rates remain below the national goal of 80% (58.5% in 2021) [2]. Research shows that parent acceptability of the HPV vaccine is largely driven by their beliefs regarding the vaccine’s effectiveness, safety, and ease of access [3]. In response to the challenges surrounding HPV vaccination, it is important to explore communication strategies to improve parental vaccination rates. One of such is the use of narratives for health promotion [4]. This study seeks to examine the role of narrative and non-narrative messages in encouraging parents to vaccinate their children against HPV. Guided by the transtheoretical model [5] and construal level theory [6], we also investigate whether the relative message effect is contingent upon parents’ readiness to vaccinate, which tends to influence their behavioral choices. We explicate the promise of matching message features and audience characteristics in terms of construal level to enhance persuasive effects.

### Narrative persuasion

Scholars have proposed various definitions of narratives [e.g. 7–10]. Despite the differences, a common theme running through them involves problem solving, interpersonal relations, and human experience [11], which concerns a representation of events and characters [7]. In health communication, narratives are in various forms, such as anecdotes, conversations, testimonials, public service announcements, and entertainment education [4]. In contrast, non-narratives typically employ an expository and didactic communication style, often with statistical evidence and logical reasoning [12].

Several theoretical perspectives explicate the power of narratives to influence health-related attitudes and behaviors. For instance, narratives provide a basis for observational learning [13]. People learn to behave by observing models, such as through exposure to narrative displays of them. In addition, narratives help to overcome resistance to persuasion [14, 15]. When people are exposed to narratives, their cognitive capacity is preoccupied with characters and events, thereby reducing counterarguing [16]. Furthermore, narratives facilitate attention and comprehension [17], particularly among individuals who have relatively lower health literacy, tend to mistrust health providers, and have limited ability and motivation to process health messages [10]. Despite the potential of narratives for health promotion, accumulated empirical evidence from health communication meta-analyses indicates inconsistent advantages over non-narratives [see e.g. 18–20]. These findings suggest that narrative effects may be contingent upon moderating factors. Therefore, the present study seeks to expand the literature by exploring narrative persuasion in conjunction with an important individual difference—readiness to change behavior.

### Stages of change: parents’ readiness to vaccinate their child

The transtheoretical model (TTM), also known as the stages of change approach, posits that behavior change unfolds over time and involves progression through five distinct stages, not necessarily linearly [5]. These stages include precontemplation (no intention to change), contemplation (intention within 6 months), preparation (intention within a month), action (behavior change within the past 6 months), and maintenance (behavior change for at least 6 months). The core idea of TTM concerns one’s readiness to change their behavior. Given that the target population in the present study comprises parents who have not begun any dose of the HPV vaccine series, we focus on the precontemplation, contemplation, and preparation stages.

In the context of the present study, a relevant concept for understanding parental attitudes toward HPV vaccination is vaccine hesitancy, defined as ‘delay in acceptance or refusal of vaccination despite availability of vaccination services’ (World Health Organization, 2015) [21]. Prior research has identified multiple types of parental attitudes toward vaccination, which generally fall into three broad categories: vaccine resistance, vaccine hesitancy, and vaccine acceptance [22, 23]. These categories align closely with the first three stages of TTM. Considering parents’ varying attitudes toward vaccination on a continuum, at one end are vaccine-resistant parents who decide not to vaccinate their child [also referred to as vaccine refusal; 24]. These parents tend to have fixed beliefs and remain impervious to recommendations [25], corresponding to the *precontemplation* stage. Moving along the continuum, vaccine-hesitant parents express doubt and uncertainty about vaccinating their child. These parents often harbor concerns about vaccine safety and effectiveness [26] and are considered in the *contemplation* stage. At the other end of the continuum are vaccine-acceptant parents who are planning to vaccinate their child. These parents typically recognize the importance of childhood vaccination and feel confident in their decision [27], placing them in the *preparation* stage.

This approach captures the continuum of vaccine attitudes—resistance, hesitancy, and acceptance—grounded in the theoretical framework of stages of change. It also emphasizes the importance of tailored communication strategies to address the unique needs of parents at each stage to encourage vaccination uptake. As individuals exhibit different levels of readiness to change, their perceptions of behavior change and responses to health messages will be different. In the next section, we explain the potential of using narrative versus non-narrative messages to enhance health communication among individuals who exhibit varying degrees of readiness to engage in behavior change.

### Interaction of stages of change and narrative persuasion

Construal level theory [6] provides a theoretical basis for understanding how people perceive behavior change and respond to persuasive messages. According to this theory, the psychological distance to an event influences people’s mental construal of the event. A psychologically distant event occurs in a remote place (spatially distant), in the distant future (temporally distant), to someone unlike oneself (socially distant), or with a low probability (hypothetically distant). People tend to perceive a psychologically distant event with an abstract and schematic mindset (i.e. high-level construal) while describing a psychologically proximate event with a concrete and detailed mindset (i.e. low-level construal). The influence of psychological distance on one’s mental construal has implications for decision-making research. That is, individuals favor perspectives that align with their current mental construal of an event [6, 28]. Below, we further elaborate on how construal levels are associated with stages of change and narrative persuasion.

#### Construal level of different stages of change

Translating the effects of psychological distance to the understanding of stages of change, we argue that one’s different readiness to change entails varying degrees of mental construal. For people who are more ready to change behavior, like those in the *preparation* stage, behavior change is assumed to be a psychologically proximate event, more likely to happen, and expected to take place sooner. In the present study context, these parents tend to consider behavior change as more specific and have a concrete plan of action in mind. This type of cognitive orientation is in line with low-level construal [29, 30]. In comparison, for people who are not ready to change behavior, like those in the *precontemplation* stage, behavior change is considered a psychologically distant event, less likely to happen, or anticipated to happen in the far future. For these parents, the idea of vaccinating their child is abstract and may be far removed from their current reality and direct experience, which is in line with high-level construal [29, 30]. As to those in the *contemplation* stage who do consider behavior change but have not taken important steps yet, the decision to vaccinate their child may be seen as a somewhat vague possibility, and their cognitive orientation may be somewhere in between low- and high-level construal.

Research has supported the arguments explicated above. For example, when thinking about temporally distant behaviors, individuals produced more general beliefs (i.e. the ‘why’ or desirability beliefs), in line with high construal; conversely, when considering behaviors in close temporal proximity, individuals formulated more concrete beliefs (i.e. the ‘how’ or feasibility beliefs), consistent with low construal [31]. Moreover, consumers who were in the predecisional phase of decision-making considered actions more abstractly while those in the post-decisional phase construed actions more concretely [30]. Taken together, one’s different stages of change entail different levels of mental construal.

#### Construal level of narrative and non-narrative messages

As suggested by construal-level theory, narrative and non-narrative messages can be conceptualized to align with different levels of mental construal [32]. As articulated earlier, narratives are often based on anecdotal evidence and personal cases. These elements are concrete representations, leading to a more emotionally involved message exposure [9]. Thus, the narrative format is specific and contextualized, which is expected to align with a low level of construal [32]. In comparison, non-narratives typically include expository arguments based on reasoning from aggregate data as opposed to individual experiences. These components are general representations with a relatively broad focus. Thus, the non-narrative format is abstract and decontextualized, which is consistent with a high level of construal [32].

The alignment between narrative/non-narrative messages and low/high construal has received empirical support. For example, Yan and Sengupta [33] compared the roles of different information formats in health risk assessment. They found that case-risk information (idiosyncratic information about a target person, like narratives) generated higher risk assessment when the judgment is psychologically close (i.e. low construal). In contrast, base-rate information (general information about a target population, resembling non-narratives) contributed to greater risk assessment when the judgment is psychologically distant (i.e. high construal). In addition, Kim and Nan [32] found that narratives led to stronger persuasive effects when combined with a present message frame (i.e. low construal); however, non-narratives yielded more favorable persuasive effects when paired with a future message frame (i.e. high construal). In sum, narratives contain features that are consistent with low construal while non-narratives have features that align with high construal.

#### Matching stages of change and message format

Considering the role of construal level in message effects research, Lee [34] postulated that matching the construal level across message components (i.e. message topic, message design, and individual message processing style) can lead to increased message effectiveness. Given that different stages of change are characterized by different construal mindsets, people may respond to health promotion messages in different ways depending on their current stages. In the present study, one useful strategy to enhance message effects is to match people’s readiness to change and message format in terms of construal level. Such alignment has the potential to formulate a coherent mental representation and increase message processing fluency, thus enhancing persuasive outcomes [34]. In particular, we argue that for people who are ready to engage in behavior change (i.e. those in the preparation stage), narrative (vs. non-narrative) messages are more persuasive. This is because a stronger readiness to change behavior and a concrete representation of information align with each other, as both reflect a lower level of construal. In contrast, for people who are not ready to change their behavior (i.e. precontemplators), non-narrative (vs. narrative) messages are more persuasive. This is because a weaker readiness to change behavior aligns with a general representation of information, as both entail a higher level of construal. As to people who are thinking about the possibility of behavior change (i.e. contemplators), narrative and non-narrative messages may demonstrate relatively equal persuasive effects. Taken together, we propose the following hypotheses and research question in the context of encouraging parents to vaccinate their child against HPV:

**H1**: There will be an interaction effect between message format (narrative vs. non-narrative) and individuals’ stages of change (precontemplation, contemplation, and preparation) on behavioral intention, such that:

**H1a**: For individuals who are ready to engage in behavior change (i.e., those in the preparation stage), narrative (vs. non-narrative) messages will lead to stronger intentions to get the HPV vaccine for their child.

**H1b**: For individuals who are not ready to engage in behavior change (i.e. precontemplators), non-narrative (vs. narrative) messages will lead to stronger intentions to get the HPV vaccine for their child.

**R1**: For individuals who are ambivalent about behavior change (i.e. contemplators), will narrative and non-narrative messages lead to similar levels of intentions to get the HPV vaccine for their child?

## Method

### Design and procedure

This study employed a single-factor (message format: narrative vs. non-narrative) between-subjects design as part of a larger project evaluating a Twitter-based HPV vaccination intervention. Participants were recruited through Qualtrics Panels from March to July 2022, when the social media platform X was still called Twitter. Eligibility criteria included: (i) being ≥18 years, (ii) using Twitter, (iii) having a child aged 9–14 years who had not started the two-dose HPV vaccination series, and (iv) not having a child who had completed any dose of the HPV vaccination series. Participants (*N* = 593) were asked to provide responses to questions evaluating their baseline stages of change along with other variables that were part of the larger project. Then, they were randomly assigned to either the narrative or the non-narrative condition and exposed to a series of Twitter threads, with each thread ranging from 4 to 8 Twitter posts on HPV vaccination in its corresponding message format. After the stimulus exposure, participants responded to items assessing intentions to vaccinate their child.

### Stimulus materials

In this study, we defined and operationalized narratives as conversation-based, character-driven exchanges between parent personas that reflect personal experiences and evoke emotional engagement. In comparison, non-narratives presented relatively expository and straightforward information. In both conditions, we provided similar health education materials and mapped seven content areas about the HPV vaccine, including normative beliefs, knowledge and awareness, access, safety, gender, disparities and equity, and cancer prevention. We strived to incorporate a relatively equal number of photos, visuals (such as GIFs), and links across the experimental conditions. Although the narrative and non-narrative Tweets were not matched strictly for length or numeracy, the narrative messages were designed to reflect naturalistic, engaging storytelling, while the non-narrative messages focused on factual clarity and straightforward presentation, with the goal of prioritizing ecological validity.

Specifically, the narrative condition featured a series of dialogues between parent personas discussing their personal experiences, concerns, and decisions regarding HPV vaccination. For example, one narrative tweet thread read, ‘[Parent 1] I’ve read that 85% of people in the U.S. will have HPV at some point in their lives. But then I also read that it usually goes away on its own. So, what’s the story? Why do we even need the vaccine? [Parent 2] That’s a question many parents have. I used to wonder the same thing. I’ve learned that while most people get HPV and rarely have symptoms, there’s no way to know which people who get HPV will develop cancer.’ These materials were developed through a community-based, formative research process that involved creating a parent advisory board and conducting focus groups with parents. The advisory board, composed of parents and caregivers of children aged 9–14 years, provided iterative feedback throughout the development and refinement of the materials. The study team created parent personas based on real-life parent experiences and insights from the advisory board. Detailed information about the development of the personas is available elsewhere [35]. This collaborative process ensured that the narrative messages were pertinent, engaging, and comprehensible to parents.

In contrast, the non-narrative condition presented expository information. For example, ‘Who gets HPV? Up to 85% of people in the U.S. will have HPV at some point in their lives. There’s no cure and some strains may go away on their own. The good thing is we can prevent HPV cancers with a 2-dose vaccine for kids ages 9–14.’ The materials were sourced from existing materials such as social media posts from federal and national agencies, including the Centers for Disease Control and Prevention, National Cancer Institute, American Academy of Pediatrics, and American Cancer Society. Sample posts from both conditions can be found in Appendix A.

All materials were qualitatively pretested with the parent advisory board. The pretest questionnaire included items related to general thoughts, message wording, image/graphic inclusion, topic importance, trust in information sources, whether the message communicated information that they would want to communicate to other parents, and general feelings and sentiments after viewing the messages. Close collaboration with the parent advisory board ensured that narrative messages were perceived as more story-like, while non-narrative messages were more generalized. In addition, these efforts also enhanced comparability between the two message formats in terms of clarity, engagement, and persuasiveness. As such, a formal manipulation check was not included in the main study to minimize reactivity effects, where participants’ awareness of the experimental manipulation might alter their responses [36]. More importantly, working with parents helped ensure that the messages in both conditions closely resembled those that parents would likely encounter in the natural social media space.

### Participants

Participants in the study are roughly evenly split between males (51.3%) and females (48.7%). Their age ranged from 25 to 72 years (*M* = 40.71, *SD* = 7.33). Most of the participants identified themselves as White (90.1%), followed by Hispanic (9.4%), Black/African American (7.8%), Asian (1.7%), and American Indian or Alaska Native (1.5%). About 9.3% of the participants reported having completed high school/GED, 17.3% having attended technical/trade school or some college, 32.5% having completed college, and 39.5% having completed graduate education. Participants income ranged from 5.1% earning less than $25 000, 13.2% earning between $25 000 and $49 999, 16.5% earning between $50 000 and $74 999, 27.2% earning between $75 000 and $99 999, 10.5% earning between $100 000 and $124 999, 15.9% earning between $125 000 and $149 999, and 11.6% earning $150 000 or more.

## Measures

### Stages of change

Following prior research [e.g. 37, 38], participants were asked to select one among five statements that best represents their readiness to vaccinate their child against HPV. Those who had ‘decided not to get my child vaccinated’ or ‘have not thought about it’ were grouped as being in the *precontemplation* stage (*n* = 102). Participants who were ‘considering it’ were in the *contemplation* stage (*n* = 265). Finally, those who were ‘planning to have my child vaccinated’ or had ‘made an appointment to have my child vaccinated’ were categorized as being in the *preparation* stage (*n* = 215).

### HPV vaccine intention

We assessed parents’ intention to get their child the HPV vaccine by asking ‘How likely are you to get your child the HPV vaccine the next time you see your child’s doctor?’ Responses were on a 5-point Likert scale ranging from 1 (*Definitely will NOT get the HPV vaccine for my child*) to 5 (*Definitely will get the HPV vaccine for my child*) (*M* = 4.24, *SD* = 1.02).

## Analysis

Before testing the main hypotheses, randomization checks showed that no demographic variables differed significantly across the experimental conditions. Thus, no covariates were included in the model. To examine the outcome variable, intention to vaccinate, a Generalized Linear Model (GLM) with a Poisson distribution and a log link function was employed. The model’s predictors included the experimental condition (narrative and non-narrative) and stages of change (precontemplation, contemplation, and preparation). Although the outcome variable was recorded on a 5-point Likert scale, typically considered ordinal, it was analyzed as discrete counts to appropriately capture increments of agreement or levels of intention. This analytical approach allowed for the inclusion of categorical predictors and yielded interpretable parameter estimates, enhancing the understanding of the associations between vaccination intentions and various predictors. To control for potential Type I errors in multiple comparisons, a Bonferroni correction was applied during follow-up tests.

## Results

In our analysis, preliminary diagnostics indicated significant underdispersion, with a deviance/DF ratio of 0.21. To ensure that the model adhered to the assumptions of the Poisson distribution, a scale adjustment was implemented to normalize this ratio to 1.00. This adjustment helps ensure the statistical tests are based on more reliable standard error estimates. The main effect of message format was significant, χ^2^(1) = 10.97, *P* < .001. The adjusted mean count for the non-narrative condition was 1.42 (*SE* = 0.02), compared to 1.32 (*SE* = 0.02) for the narrative group, meaning that parents who were exposed to non-narrative messages were more likely to take action to vaccinate their children than those exposed to narrative messages. The main effect of stages of change was also significant, χ^2^(2) = 107.94, *P* < .001. Specifically, those in the preparation stage reported a stronger behavioral intention (*adjusted M* = 1.53, *SE* = 0.01) than contemplators (*adjusted M* = 1.48, *SE* = 0.01), *P *= .005, and precontemplators (*adjusted M* = 1.10, *SE* = 0.04), *P *< .001. In addition, the difference in behavioral intention between contemplators and precontemplators was also significant, *P *< .001.

Moreover, the interaction effect between message format and stages of change was significant, χ^2^(2) = 7.68, *P* = .02. To address our hypotheses, we chose to focus exclusively on the simple main effects of stages of change and analyze how narrative and non-narrative formats influenced each stage separately. H1a predicted that narrative (vs. non-narrative) messages would generate a stronger behavioral intention among those in the *preparation* stage. However, we did not find any significant differences between the two message formats in influencing behavioral intention. Thus, H1a was not supported. H1b predicted that non-narrative (vs. narrative) messages would be more effective in enhancing behavioral intention among people in the *precontemplation* stage. Consistent with the prediction, we found that non-narrative (*adjusted M* = 1.22, *SE* = 0.06) generated a significantly stronger behavioral intention than narratives (*adjusted M* = 0.97, *SE* = 0.06) among precontemplators, χ^2^[1) = 9.21, *P* = .002, supporting H1b. To address R1 regarding the message effects among individuals in the *contemplation* stage, results showed that message formats did not have a significantly different impact on behavioral intention among contemplators.

## Discussion

### Theoretical implications

This study sheds light on the literature on narrative persuasion and stages of change in several ways. First, the findings show that the effects of narrative and non-narrative messages vary across stages of change. Contrary to the assumption that narratives universally outperform non-narratives, the findings suggest that their relative advantage depends on the audience’s readiness to engage in behavior change. In particular, for individuals who are not ready to take action, non-narratives are more effective in bolstering behavioral intention. However, for individuals who are considering or planning for behavior change, the two message formats demonstrated similar effects on behavioral intention. These findings suggest that while narratives hold promise for influencing health behavior, it is crucial to consider how these messages function under different conditions. The findings also echo the implications from the existing meta-analyses suggesting that the effects of narrative persuasion are contingent upon moderators, a line of research that future studies should continue exploring.

Moreover, the findings allude to the usefulness of construal level theory in guiding message effects research. As mentioned earlier, Lee [34] proposed that matching the construal level across message components has the potential to enhance message effects and persuasive outcomes. We observed patterns that are consistent with this proposition, such as the relative advantage of non-narrative messages among precontemplators. However, it is important to note that our study did not directly measure or test construal level constructs. Future research could explicitly measure psychological distance or construal levels to further examine their roles in moderating the effects of narrative and non-narrative messages. It is also important to highlight the possibility of alternative theoretical reasoning in explaining this finding. The conversational structure embedded in the narratives might have been interpreted as endorsing the HPV vaccine and conveying social norms, thus inadvertently inciting a threat to freedom. The non-narrative message, however, may lack elements that conspicuously elicit a threat to freedom.

Contrary to the prediction, we found that narrative and non-narrative messages did not demonstrate significantly different impacts on behavioral intention among those who are more ready to engage in behavior change (i.e. those in the preparation stage). This finding suggests that there could have been a ceiling effect for people who are already motivated to change their behavior. Parents who are more ready to take action may have taken important steps toward behavioral modification. Therefore, how information in the message is conveyed may not make a difference, and both message formats may serve as effective reminders.

### Practical implications

The present research also offers several practical implications for health message design. First, health message designers should recognize that narratives may not always provide a persuasive advantage, particularly for audiences at earlier stages of readiness to change. Instead, message designers may take advantage of the power of non-narrative messaging. Although narratives are a compelling way to evoke emotions and enhance persuasion [39], non-narratives may resonate with people who view behavior change as psychologically distant. This may be because non-narratives are typically brief, straightforward, and easy to understand in a fast-paced media environment. Additionally, unlike traditional didactic communication, non-narratives on social media are often designed to be appealing and accessible with visual elements, such as the infographics used in the present study. Such creative design elements can help deliver information and capture attention.

Furthermore, the findings emphasize the importance of considering message tailoring and audience segmentation in health message design. Our findings suggest that when the target audiences are not ready to take action, health promotion practitioners may use non-narratives to promote behavior change; whereas for the target audiences who are thinking about or motivated to engage in behavior change, both message formats would be effective tools to reach people and enhance persuasion.

### Limitations and directions for future research

This research has several limitations. First, parents on Twitter (now known as X) may not be representative of parents on other social media platforms or who do not use social media. Also, the sample in the study consisted of participants who have a relatively higher educational level. They may be more comfortable with interpreting health information, regardless of the format. Second, the information exchanged in the narrative condition among parent personas does not always have a direct counterpart in the non-narrative condition, and we did not conduct a quantitative pretest to statistically validate differences between the two. Potential confounding variables, such as message length, informativeness, content variation, and perceived message interactivity, may have contributed to the observed effects. While achieving exact equivalency across conditions is challenging, we prioritized ecological validity by designing messages for a naturalistic setting and refining them through an iterative, community-based approach. Future research should incorporate a quantitative pretest to validate differences in narrativity. Third, stages of change and vaccination intention were assessed using single-item measures, which may not capture the full complexity or nuance of these constructs. Future studies should consider employing multi-item scales to provide a more comprehensive assessment of these constructs. Fourth, we examined participants’ immediate, initial reactions to the messages. It is unknown whether the observed effects will persist in the long term. Future research with a longitudinal design that traces the actual behavior change may help substantiate the influence of narrative and non-narrative messages on behavior change.

## Conclusion

In this study, we examined the promise of matching the construal level between narratives/non-narratives and stages of change to enhance message effects. In the context of encouraging parents to vaccinate their children against HPV, we found that non-narratives are more effective in bolstering behavioral intention than narratives among individuals who are not ready to engage in behavior change. For those who are thinking about and motivated to take action, both message formats were effective. We hope future research can build on these findings and continue the investigation of message components with synergetic functions in enhancing health message effects.

## Figures and Tables

**Fig 1.**
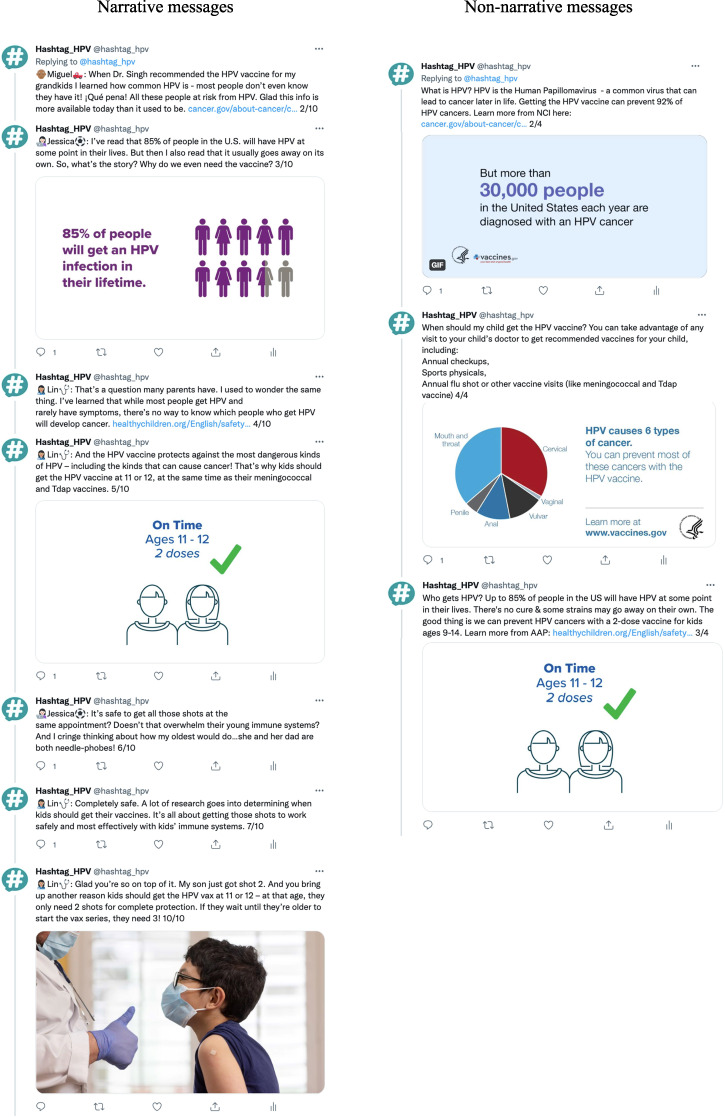
Narrative messages and Non-narrative messages
